# Thermal Stability and Dynamic Mechanical Analysis of Benzoylation Treated Sugar Palm/Kenaf Fiber Reinforced Polypropylene Hybrid Composites

**DOI:** 10.3390/polym13172961

**Published:** 2021-08-31

**Authors:** S. Mohd Izwan, S.M. Sapuan, M.Y.M. Zuhri, A.R. Mohamed

**Affiliations:** 1 Centre of Advanced Engineering Materials and Composites Research, Department of Mechanical and Manufacturing Engineering, Universiti Putra Malaysia, UPM, Serdang 43400, Malaysia; mr_iez88@yahoo.com (S.M.I.); zuhri@upm.edu.my (M.Y.M.Z.); 2 Laboratory of Bio Composite Technology, Institute of Tropical Forestry and Forest Products, Universiti Putra Malaysia, UPM, Serdang 43400, Malaysia; 3 Department of Manufacturing and Material Engineering, Kulliyyah of Engineering, International Islamic University Malaysia, Kuala Lumpur 50728, Malaysia; mrahman@iium.edu.my

**Keywords:** biocomposites, kenaf, sugar palm, thermal, dynamic mechanical analysis, benzoylation

## Abstract

This research was performed to evaluate the mechanical and thermal properties of sugar palm fiber (SPF)- and kenaf fiber (KF)-reinforced polypropylene (PP) composites. Sugar palm/kenaf was successfully treated by benzoylation treatment. The hybridized bio-composites (PP/SPF/KF) were fabricated with overall 10 weight percentage (wt%) relatively with three different fibers ratios between sugar palm-treated and kenaf-treated (7:3, 5:5, 3:7) and vice versa. The investigations of thermal stability were then carried out by using diffraction scanning calorimetry (DSC) and thermogravimetry analysis (TGA). The result of a flammability test showed that the treated hybrid composite (PP/SPF/KF) was the specimen that exhibited the best flammability properties, having the lowest average burning rate of 28 mm/min. The stiffness storage modulus (E’), loss modulus (E”), and damping factor (Tan δ) were examined by using dynamic mechanical analysis (DMA). The hybrid composite with the best ratio (PP/SPF/KF), T-SP5K5, showed a loss modulus (E”) of 86.2 MPa and a damping factor of 0.058. In addition, thermomechanical analysis (TMA) of the studies of the dimension coefficient (µm) against temperature were successfully recorded, with T-SP5K5 achieving the highest dimensional coefficient of 30.11 µm at 105 °C.

## 1. Introduction

For decades, synthetic fibers have been the leading commodity in the composites industry. However, synthetic fibers possess many disadvantages, as they catch fire easily very hydrophobic and non-biodegradable. Since synthetic fibers have many shortcomings, researchers have had growing interest in producing polymers that incorporate natural fibers. Natural fibers are becoming more common as a viable option due to the harmful environmental and health consequences of synthetic fibers. Concerns about the environment and the rising greenhouse effect and increasing interest in the use of sustainable materials has motivated researchers to investigate biocomposite materials. In today’s manufacturing environment, natural fiber composites are playing a prominent role in many vital applications, such as in fuselages and propellers in the aerospace industry, racing car bodies, wings of wind turbines, bicycle frames, and automobile interiors, seat cushions, and door panels, etc. [1,2,3]. The great interest in natural fiber composites is due to their high performance, biodegradability, non-abrasive light weight, and low cost [4,5]. Moreover, the widespread adoption of natural fibers and biopolymers as green materials is being motivated by the rapid depletion of petroleum supplies, as well as by a growing recognition of global environmental issues associated with the use of traditional plastics. [6,7,8]. The successful application of biopolymers and the promise of alternative pathways with a reduced carbon footprint arising from the use of green materials bodes well for the future design and development of ever more sophisticated green materials [9].

Natural fibers and biopolymers have attracted scientists and industry because of their environmentally beneficial and long-lasting properties. Natural fibers such as sugar palm fiber, corn husk fiber [10], jute, and wheat arrowroot, as well as cassava bagasse, are used as reinforcement materials in polymer composites for a variety of reasons, including their ability to be reusable and their low cost, and because they are environmentally sustainable and have good strength and stiffness properties [11]. For material applications, a broad variety of naturally occurring biopolymers extracted from renewable materials are available. Some bacteria and plants (chitin, starch, and cellulose) are currently used in commercial products, whereas others are underutilized [12]. Starch has been explored as a possible alternative to traditional plastic packaging. These starch (or cellulose) biopolymers include animal-based (chitin) polymers and microbial (exopolysaccharides and polyhydroxyalkanoate) polymers [13] that are chemically synthesized from agro-based resource monomer (poly lactic acid) as well as chemically synthesized from conventionally synthesized monomers. Despite their current use, starch biopolymers have been characterized as having weak mechanical properties and a low water barrier resistance [14,15]. These drawbacks have significantly hindered a wider range of their application, especially in packaging [16,17]. Much research has been performed in an attempt to improve the mechanical properties of starch biopolymers without affecting their biodegradation properties. Research has found that reinforcing starch biopolymer with natural fiber is one way to strengthen both its mechanical and thermal properties.

Among the many different types of natural resources, kenaf plants have been extensively exploited over the past few years [18]. One of the reasons for this growing interest is that natural fibers such as kenaf have a higher specific strength as glass fiber and a similar specific modulus strength [19]. Kenaf, which is from the Hibiscus cannabinus family, was selected due to its low cost, low density, good toughness, recyclability, good sound absorption performance, acceptable strength properties, and biodegradability [20,21]. Furthermore, kenaf fiber was selected because it has already been utilized in the automotive industry and because it has a good surface and produces a lightweight material with high mechanical properties and thus does not have to prove itself as a reliable product. Since kenaf brings a lot of promising qualities, a study on the hybridization of two types of natural fiber-reinforced thermoplastic composites was an alternative option for a novel breakthrough. The hybridization of composite fibers refers to the merger of two or more reinforcing materials, such as filler, to enhance the overall properties of a material [22].

Meanwhile, sugar palm (also known as Arenga pinnata) is a tropical tree that belongs to the Palmae family. Apart from the production of its sugar and starch extract [23], this tree was also known for the fiber from its trunk and from its fronds: sugar palm fiber (SPF). Traditionally, sugar palm fiber was used for various domestic materials [24]. This is due to the excellent characteristics of sugar palm fiber that improve tensile strength and reduce the wettability degree of a composite surface. Owing to that, incorporation of treated fibers in a polymer matrix promised a good thermal resistance and reduced thermal degradation [25]. As the research has grown, sugar palm fiber has shown many significant advantages to be considered: it is abundant and widespread, and it shows a promising ability to enrich physical and mechanical strength, thermal stability, and density, as well as showing excellent biodegradability [26].

The hybridization of sugar palm fiber and kenaf fiber as a filler was not a recent finding for composite reinforcement. Polymer composites with reinforced fibers usually consist of more than one type of particle fiber compounded together with a polymer as part of their matrix system [27]. The properties of a hybrid composite are influenced by the fiber content, length, and orientation. The selection of the fiber constituent for hybrid composites affects the hybridization and the requirement of the material being fabricated.

Several studies have shown that hybridization of natural and synthetic fiber can improve mechanical and thermal properties. A study by Devi et al. [28] showed that the dynamic mechanical properties—including the storage modulus, loss modulus and tan δ—of pineapple/glass hybrid-reinforced unsaturated polyester resin composites were enhanced when more content of glass fiber was added to the composites. A previous study examined the effects on a composite’s behavior of combining rattan nanoparticles into polypropylene with filler contents ranging from 2% to 20% [29]. The study found that 5% was the most optimal filler content for achieving better mechanical properties of the composite. Furthermore, mechanical performance decreased when the filler content was increased from 5% to 20%. Another research study observed hybrid composites with different composition that were prepared with different amounts of fibers (i.e., 10%, 20% and 30% by weight percent), in which the ratios between sugar palm and kenaf fiber were 30:70, 50:50, and 70:30. The study found that the tensile strength of composites tended to decrease when the content of loading fibers increased [30]. Therefore, the current study focused only on the implementation of 10% of kenaf/sugar palm as the composite filler content in order to offer better bonding between the fibers and polypropylene matrix.

The selection of compatible fibers and fiber properties, therefore, contributes a critical aspect in designing a better hybrid composite. The previous research has thoroughly examined the effects of benzoylation treatment of SPF with different parameters. A previous study found that kenaf and sugar palm fibers were compatible combinations for hybrid composites due to the outcomes of high tensile strength and toughness of the kenaf/sugar palm composites [31,32,33]. As reported, the benzoylation of fibers improves fiber–matrix adhesion, thus improving thermal stability, increasing composite strength, and decreasing water absorption [34,35,36]. Benzoyl chloride was used in this research for SPF and KF treatment. This benzoyl chloride helps to decrease the hydrophilic nature of SPF and KF and improves the interaction with the resin matrix [37].

Hence, in this paper, the hybridized polypropylene composite with kenaf/sugar palm fiber was further examined for its thermal stability using dynamic thermal analysis. The preparation and characterization of thermosetting and thermoplastic composite materials reinforced with kenaf and sugar palm fibers with and without treatment using benzoylation methods were conducted. As presumed, treatment using alkaline on the surface of the fiber changed the surface wettability, altering the mechanical and physical properties of the natural fibers. Moreover, the benzoylation of kenaf and sugar palm fibers treatment also successfully confirmed an incremental increase in tensile strength. Thus, the main objective for this paper was an investigation of thermosetting composites based on their thermal stability, thermal degradation, flammability, and modulus stress by using instrumentation such as diffraction calorimetry (DSC), thermogravimetry analysis (TGA), dynamic mechanical analysis (DMA), thermomechanical analysis (TMA), and flammability analysis.

## 2. Material and Methods

### 2.1. Materials

Polypropylene pellet and benzoyl chloride were supplied by Mecha Solve Engineering (M) Sdn Bhd. Sugar palm fiber (SPF) was purchased from Jempol, Negeri Sembilan, Malaysia, and kenaf fiber (KF) was obtained from Lembaga Kenaf and Tembakau Negara (LKTN) Kelantan, Malaysia. Kenaf (*Hibiscus cannabinus*) and the sugar palm tree (*Arenga pinnata*) were used. In addition, raw kenaf palm and kenaf fibers were washed with deionized water and rinsed. They were then pulverized, cleaned, and dried at 70 °C in an oven. All other chemicals and solvents that were used in this work were at 98% purity. Polypropylene pellet crystals with 0.946 g/cm^3^ density were used. The pellets that were used were whitish gray, ovular, and 5mm long and 3mm diameter.

### 2.2. Alkalization and Benzoylation of Kenaf and Sugar Palm Fiber (KF/SPF)

The clean dried kenaf and sugar palm fibers were then soaked. An amount of this fiber was soaked in 18% concentration of NaOH solution as a pre-treatment for 30 min. After that, the partially treated kenaf and sugar palm fibers were filtered and rinsed with ionized water and dried in an oven at 70 °C [34,35]. The treated fibers were then immersed in 10% concentration NaOH solution agitated well with 50 mL benzoyl chloride for 15 min. The treated KF and SPF was then soaked with ethanol for 1 h and rinsed with tap water in order to remove unreacted benzoyl chloride and excess dirt. Treated KF and SPF were then dried at 60 °C for 24 h [36]. Alkalization of the samples was performed to remove impurities and benzoylation was performed to enhance the melting point of the samples [37].

### 2.3. Compounding of Kenaf and Sugar Palm Fibers (KF/SPF) and Preparation of Particle Composite

The treated and untreated kenaf and sugar palm fibers were ground into short-form fibers with an approximate length of 0.1–0.5 mm by using a pulverizing machine (Pulveriseet P-19). In order to obtain uniformly cut fiber particle sizes, the fibers were sieved by using a 40 mesh electronic sieve (Endecotts). Finally, ground KF and SPF fibers were dried at 60 °C for 12 h to avoid contamination. Next, a melt extruder was used to compound the treated and untreated ground KF and SPF with polypropylene as their polymer matrix by using a Brabender plastograph (Model 815651, Brabender GmbH & Co. KG, Duisburg, Germany). An amount of 20 g of mixture was prepared for each cycle of extrusion, and the compositions of the hybrid composites of SPF/KF/PP are presented in Table 1. The initial ‘U’ and ‘T’ indicate untreated and treated hybrid composites, respectively.

**Figure 1:**
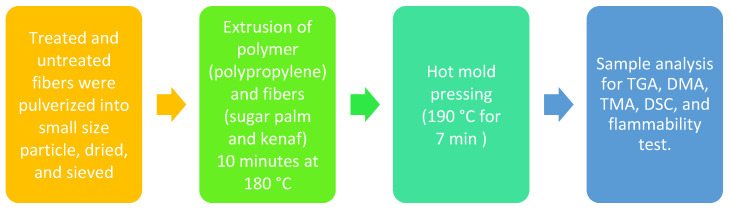
Flowchart of different steps for polypropylene with sugar palm and kenaf fibers (PP/KF/SPF) composite.

The mixing temperature was set at 180 °C, while the rotor speed of the rotating screw was set at 50 rpm. Polypropylene was discharged in the chamber and melted for 3 min before the compounding took place. The KF and SPF particle fibers and the polymer were extruded over approximately 10 min of holistic mixing. Thermoset composites of SPF/KF/PP were crushed into granular size, followed by the use of a hot mold pressing. Customized samples of hybrid composites were then pre-heated at 180 °C for 5 min and pressed at 190 °C for 7 min by using a hot press machine. After that, the composite samples were cold pressed at 25 °C for 5 min and chopped into plain composite sheets sized 150 × 150 × 3 mm before being cut into a standard shape for TGA, DMA, TMA, DSC, and flammability test, as illustrated in Figure 1.

### 2.4. Thermal Instrumentations

#### 2.4.1. Thermogravimetric Analysis (TGA and DTG)

Thermogravimetric analysis (TGA) was performed to examine the structural properties and thermal stability of the hybrid composite materials. The analysis was carried out to calculate the degradation curve of the SPF/KF/PP composites towards their degradation temperature (°C). A Mettler Toledo machine (TGA/SDTA 851e, USA) was used, and all composites (SPF/KF/PP) were observed between 30 to 600 °C at a heating rate of 20 °C/min. Nitrogen gas flow was recorded at 50 mL/min. The weight of the samples varied from between 6 and 20 mg.

#### 2.4.2. Differential Scanning Calorimetry Analysis (DSC)

Differential scanning calorimetry (DSC) analysis of the samples was carried out with a PerkinElmer (USA) Diamond thermogravimetric (TG)/DSC analyzer. The work was carried out with 20 milligram of the composite fibers sample filled in aluminum pans under a dynamic nitrogen atmosphere in a temperature range of 25–600 °C and a heating rate of 5 °C/min. The percentage of crystallinity Χ (%) was calculated as Equation (1):

(1) $$\frac{\Delta H\;}{\Delta H_{100}}=\;X^{C}$$

where ΔH is the heat of crystallization of the sample analyzed (J/g), and $\Delta H_{100}$ is a reference value that represents the heat of crystallization for a 100% crystalline polymer. For PP, $\Delta H_{100}$ is taken as 209 J/g.

#### 2.4.3. Dynamic Mechanical Analysis (DMA)

A dynamic mechanical analyzer (TA Instrument, Q800, USA) was used for the evaluation of the storage modulus, loss modulus, and mechanical damping factor (tan δ). The storage modulus (E’), loss modulus (E”), and loss factor (tan δ) of the composite specimen were evaluated as a function of temperature (−100 °C to 100 °C) using TA 2980 software (TA Universal Analysis, USA). A dynamic mechanical analyzer was equipped with a dual cantilever bending fixture at the frequency of 1 Hz with the heating constant rate at 10 °C/min. Three-point bending mode was examined. The heating rate used was at 5 °C/min under an amplitude frequency of 1 Hz. Liquid nitrogen was used as the cooling agent, and the temperature range was from −150 °C to 150 °C. The amplitude was set at 7–10 mm, depending on the thickness of the samples. The samples had a thickness of 4–5 mm, width of 9–10 mm, and length of 50–60 mm.

#### 2.4.4. Thermal Mechanical Analysis (TMA)

The coefficient of thermal expansion (CTE) was measured by heating the specimen from −50 °C to 100 °C at a rate of 5 °C/min under a nitrogen atmosphere with a flow rate of 100 mL/min. The probe was applied with a 0.05 N loading to measure the strain in the specimens and their temperature. The coefficient of thermal expansion was estimated from the linear slope of the strain–temperature curve using a thermomechanical analyzer (TMA Q 400, TA Instruments, New Castle, DE, USA). The specimen dimensions were 7 mm × 7 mm × 1.8 mm.

#### 2.4.5. Flammability Analysis

**Figure 2:**
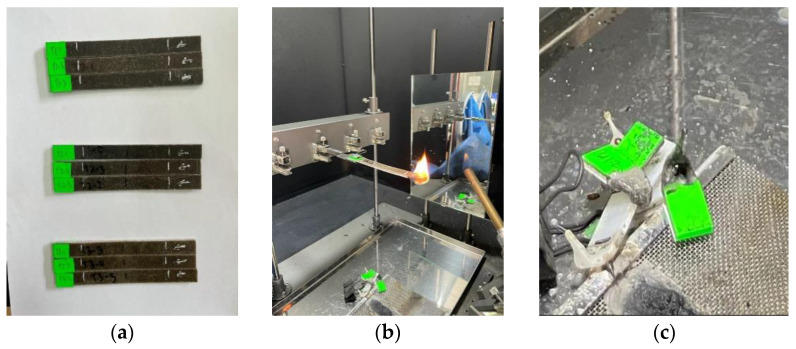
(**a**) Composite hybrid (SP/K/PP); (**b**) Composite hybrid (SP/K/PP) performance in flammability test; (**c**) Sample debris after test 3.

A flammability test of pure PP and SPF/KF/PP composites was carried out for all samples via a horizontal burning test according to ASTM D635. Three specimens from each composite ratio, with dimensions of 125 mm × 13 mm × 3 mm, were prepared, and two lines at 25 and 100 mm from one end of the sample were drawn as the reference marks as shown in Figure 2a [38]. Then, the burning time from the first reference mark to the second reference mark (25 mm from the end and 100 mm from the end, respectively) was recorded as shown in Figure 2b. The linear rate of the burning samples was calculated using Equation (2).

(2) $$V=\frac{L}{t}\;60$$

where *V* is the linear burning rate (mm/min), *L* is the burnt length (mm), and *t* is the time (minutes) [35]. 

## 3. Results and Discussions

### 3.1. Thermogravimetric Analysis (TGA and DTG)

Thermogravimetry is primarily influenced by an accurate heating rate and conditions. Thermogravimetry offers a quantifiable analysis of the amount of moisture and volatile compounds present in fibers, the weight loss, and the thermal breakdown. It also assists in determining the degradation mechanism. Thermogravimetric analysis (TGA) and derivative thermogravimetric (DTG) curves of hybrid composites of sugar palm, kenaf, and polypropylene (SPF/KF/PP) were employed to investigate the thermal stability and decomposition of the polymeric system. As shown in Table 2, weight loss (%) at T_max_ and percentage residual (%) were investigated with different composition weight ratios of hybrid samples.

**Figure 3:**
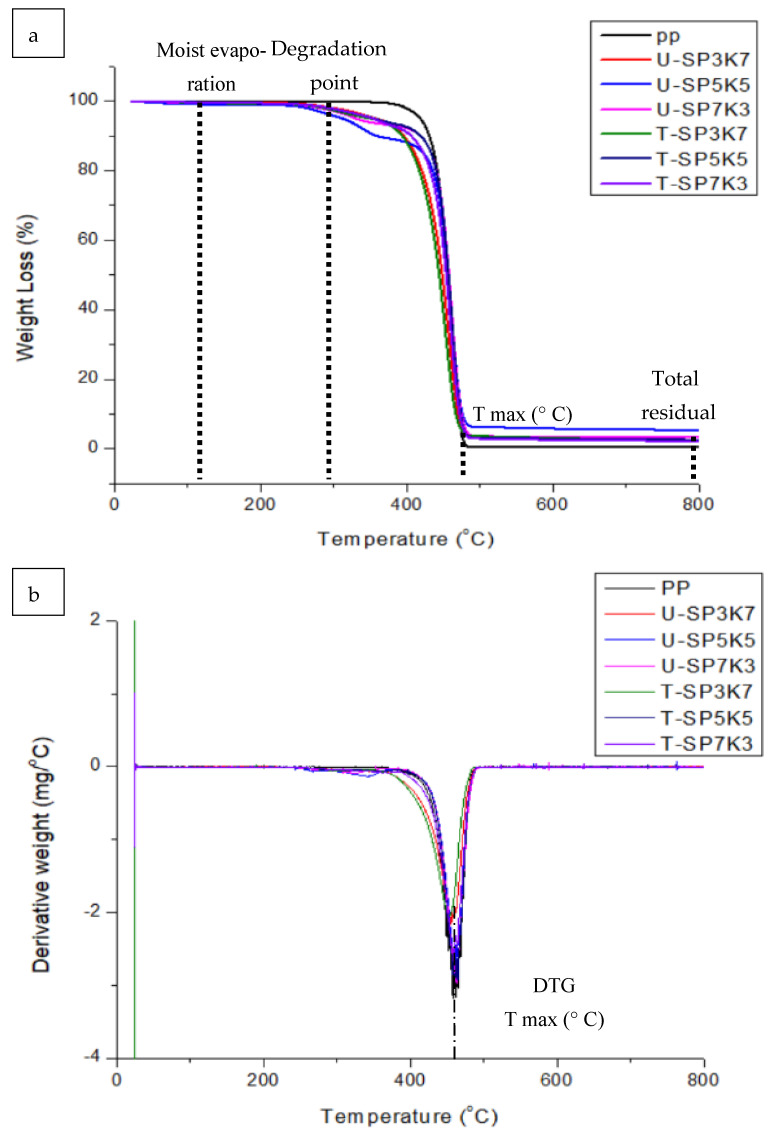
TGA composite hybrid SPF/KF/PP with varied composition: (**a**) Weight loss (%) analysis of SPF/KF/PP; (**b**) Derivative thermogravimetric (DTG) of SPF/KF/PP.

Pure PP [39], treated, and untreated fibers hybrid composite SPF/KF/PP with varied ratios were compared, as illustrated in Figure 3. The thermogram in Figure 3a shows that at the first quartile degradation, there was a slight weight loss for all SPF/KF/PP hybrid composites ratios. Owing to that, the reduction in percentage weight was due to the release of moisture content in the SPF/KF/PP hybrid composites [40]. In addition, because of the differences in the chemical topology of the fiber components—mostly lignin, hemicellulose, and cellulose—kenaf and sugar palm fibers presumed to decomposes at significant temperatures. In this study, it was confirmed that the thermal degradation of PP kenaf/SP composites had a multi-stepped degradation. The initial transition between 30 and 150 °C indicates the water loss of SPF/KF/PP hybrid composites [41].

Different natural fibers exhibit different decomposition profiles, as shown in Figure 2. The curves for pure PP are also presented for comparison. For all SPF/KF/PP hybrid composite formulations, the TG curves (Figure 3a) indicate that composites containing kenaf fibers and sugar palm exhibited very similar weight loss trends until the temperature range of 400–450 °C, where the second quartile of degradation took place. During the second quartile, the weight loss can be seen at max, which is when composite tends to decompose at a higher rate. This is explained by the fact that other elements inside the fiber, such as lignin, cellulosic material, and detangled of hydrogen bond of polypropylene, are decomposing [41].

As shown in Table 2, pure polypropylene showed the highest weight loss at T_max_(wt%), which can be seen at 99.46%, with only 0.53% total residual char at 800 °C. In addition, the untreated hybrid composite with 5 percent of sugar palm and kenaf fiber (U-SP5K5) showed an increment of total residual after 800 °C. However, the highest total residual of SPF/KF/PP hybrid composite T-SP5K5 was recorded at a total weight residue of 15.8% at T_max_ and 5.22% char at 800 °C. First, as shown in TGA analysis, the addition of fibers increased thermal stability by lowering the total weight loss at T_max_, which can be seen by the weight loss (%) trends at T_max_ for all compositions of the SPF/KF/PP hybrid composite. Furthermore, the benzoylation treatment gave an additional retardancy to the thermal stability [42].

Figure 3b shows the derivative thermogram (DTG) analysis. The DTG curves of the composites reveal that their degradation process occurred in three stages. Figure 3b shows zero degradation at first and the derivative weight decreasing at around 350–400 °C degradation. It starts with the decomposition of the PP, followed by that of the U-SPK and T-SPK. U-SP5K5 showed the least derivative weight loss for untreated hybrid composite. Meanwhile, the best degradation behavior of the SPF and KF hybrid composite was for T-SP5K5. The peak of degradation for all samples showed that organic elements start first to deteriorate. This is explained by the presence of moisture in the fiber and its loss at the first quartile, 100–200 °C. U-SPFKF responds to the decomposition process earlier than the T-SPFKF composites because the benzoylation treatment increased the thermal stability of SPF/KF/PP. The rearrangement of the hydroxyl group after benzoylation treatment inside the cellulosic moieties of the fibers presumes to increase the heat retardancy of the hybrid composites and to slow the thermal degradation [43].

### 3.2. Differential Scanning Calorimetry Analysis

**Figure 4:**
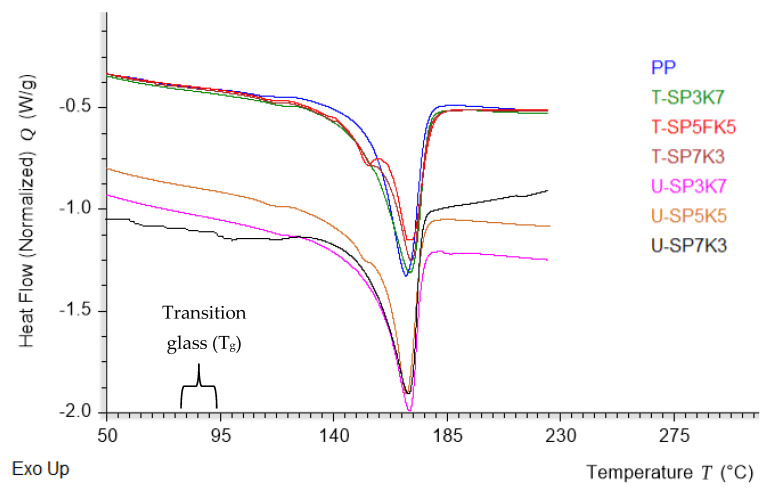
DSC curve of SPF/KF/PP hybrid composite with different composition ratios.

The DSC curves of the hybrid composites SPF/KF/PP are presented in Figure 4. Information on the DSC analysis is listed in Table 3 and discussion of the analysis follows. As observed from the graph in Figure 4, all samples shared comparable values around 95–125 °C, which was due to the loss of moisture from all composites samples. Polypropylene did not show any transition glass temperature, as it is in a crystalline state. In comparison with the treated and untreated composite SPF/KF/PP, the glass transition temperature of all hybrid composite compositions showed a slight peak of transition glass (T_g_) temperature. The transition glass temperature was shown to be the highest for the T-SP5K5 hybrid composite at 121.43 °C and a melting point at 161.43 °C, compared with the other composition ratios. According to Phiri, Khoathane, and Sadiku [44], the melting point (T_m_) of polymer PP occurs at around 146.43 °C and increases gradually after incorporation of kenaf and sugar palm fibers. Hybrid composites U-SP7K3, U-SP5K5, and U-SP3K7 showed T_m_ at 149.43, 155.63, and 148.33 °C, respectively. Table 3 also shows the results of treated sugar palm and kenaf fibers with benzoyl treatment, which possessed a better melting point and transition glass temperature.

A significant trend was shown in the results of treated kenaf and sugar palm filler with T-SP7K3, T-SP5K5, and T-SP3K7, exceeding 122.53, 127.63, 125.43 °C, respectively, for their transition glass temperature (T_g_). T-SP5K5 achieved the highest values of T_g_ and T_m_ at 127.6 and 165.63 °C, which might correspond to the additional increased interaction between matrix and filler and might have led to a restriction in the polymer chain of the composites [45]. A more noticeable effect on the thermal properties of the hybrid composites can be observed through the enthalpy peaks of the DSC curves. All peaks occurred at a T_m_ approximately in the same temperature range but at different enthalpy intensity, which took place at around 140–185 °C [46]. These results were in good agreement with the above discussion, where the effect of the benzoyl group on the surface of KF and SPF after benzoylation treatment increased the composites’ thermal endurance compared with the untreated fibers. In addition, as we have proved in the research, the existence of a T_g_ peak shows that the hybrid composite SPF/KF/PP in all composition is amorphous [47].

The ΔHc (crystallization enthalpy) values of the PP were obtained at 126.7 J/g. The result demonstrates that the ΔHc of composites decreased with the absence of both treated and untreated sugar palm and kenaf fibers. This trend is in agreement with the results of Huda et al., where lower melting temperature and crystallization enthalpies of the composites was observed to decrease with the addition of recycled newspaper cellulose fibers and talc, compared with neat PP [48]. Table 3 also demonstrates that the degree of crystallinity of SP/K/PP composites was lower compared with neat PP, which was below 60%. A significant trend was shown in the results of treated kenaf and sugar palm filler with U-SP7K3, U-SP5K5, and U-SP3K7, which had overall greater crystallinity degree at 46.63%, 57.87%, and 47.44%, compared with treated composites T-SP7K3, T-SP5K5, and T-SP3K7 with values of 53.22%, 52.88%, and 50.66%, respectively. Furthermore, the addition of fibers content in PP resulted in a decrease in the degree of crystallinity of the polymer, which happened due to both treated and untreated fillers kenaf and sugar palm fibers obstructing the mobilization of the PP macromolecular chain and preventing the macromolecular segment from obtaining an ordered alignment of the crystal lattice. Cellulose is also meant to hinder the formation of crystallinity in polymer. Thus, the crystallinity of composites was decreased [49].

### 3.3. Flammability Analysis (FA)

**Figure 5:**
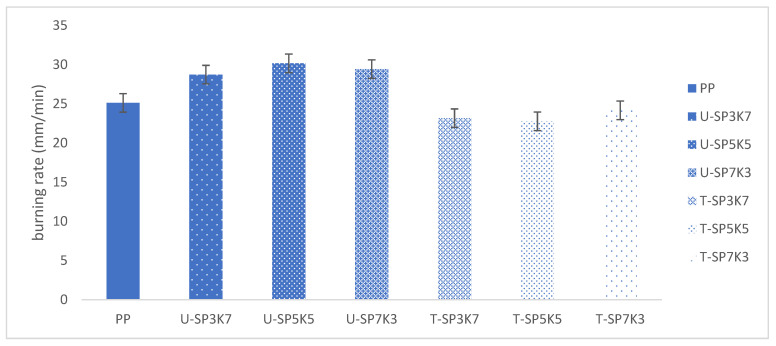
The effect of different fiber loadings on the burning rate of (SP/K/SP) hybrid composites.

One of the characterizations of plastics resin is that they can easily flare up when exposed to sufficient heat in the presence of oxygen. Because of the rate of burning for plastics, considerable work has been directed to the study and minimization of the flammability of these materials, such as by the addition of flame retardant chemicals to prevent or minimize the combustion of these materials. A test was done to classify and measure the burning characteristics of the plastics resin. The burning rates of PP and PP composites measured by a horizontal burning test are shown in a bar chart in Figure 5.

Overall, neat PP and untreated and treated kenaf and sugar palm composites U-(SPF/KF) /T-(SPF/KF) showed a burning rate (mm/min) for neat polypropylene of 25.12 (mm) min^−1^. Most polymer resins, including PP, are extremely flammable. During the burning process, the untreated kenaf and sugar palm hybrid composite formed a non-protective oil layer on the surface of the matrix, serving as an oxygen conductor and permitting heat to penetrate the matrix [50,51]. Therefore, the quantity of decomposed volatiles that escaped the interior polymer matrix was increased, resulting in a shorter burning time and thus increasing the linear burning rate.

With the incorporation of untreated kenaf and sugar palm fibers, a higher burning rate was recorded. Examination shows that composite hybrid samples U-SP3K7, U-SP5K5, and U-SP7K3 achieved 28.72, 30.16, and 29.43 (mm) min^−1^ burning rates, respectively. In these cases, natural fibers are expected to act as combustion sources for the composites. As we know, fibers have high sensitivity towards flame; thus, the incorporation of fibers indeed increased the flammability rate. The high lignin content of kenaf, as compared with some other natural fibers such as flax, hemp, and sisal [48], promotes high heat of combustion and initiates ignition by reducing the thermal stability, which promotes ignition. Generally, kenaf and sugar palm fibers, like other natural fibers, consist of 60–80% cellulose, 5–20% lignin (pectin), and up to 20% moisture [52,53].

However, with the incorporation of benzoyl treatment of palm and kenaf fibers. The flammability rate was reduced consistently. The treated hybrid composites T-SP3K7, T-SP5K5, and T-SP7K3 showed 23.16, 22.76, and 24.16 (mm) min^−1^ burning rate, respectively, which indicates that PP composite incorporating benzoyl-treated fibers has improved flame retardancy properties, compared with the untreated sample of hybrid composites [54]. In this experiment, it can be deduced that the incorporation of untreated fibers increases the burning rate of the fibers and lowers the burning rate of treated fibers.

### 3.4. Dynamic Mechanical Analysis (DMA)

**Figure 6:**
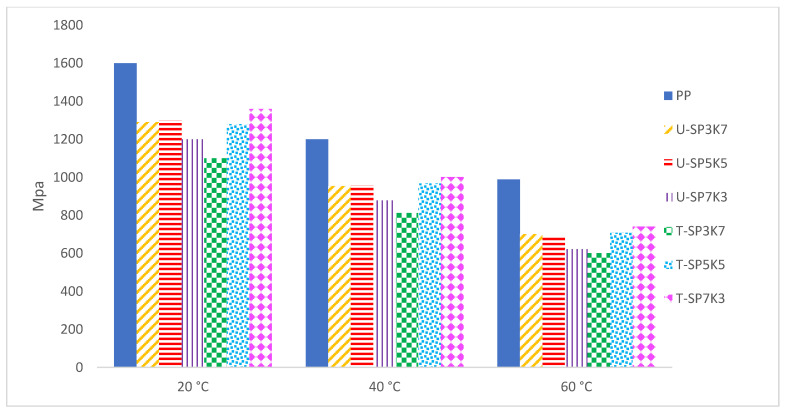
Temperature dependence of the storage moduli (E’) MPa of varied compositions SPF/KF/PP hybrid composite at 20, 40 and 60 °C.

Typically, DMA is conducted to assess differences in the stiffness, damping, and T_g_ of polymeric composites during curing [55]. A DMA exhibits the outcomes on storage modulus (E′), which is related to the Young’s modulus of the composite. The storage modulus, or E′, is exploited by material researchers to identify the stiffness of a composite. In general, the E′ describes the ability of a material/composite to store energy for the upcoming application [56]. A viscous response of a material/composite is referred to as loss modulus (E″) or dynamic loss modulus [57,58]. E″ establishes output data on the tendency of material/composites to release the applied energy, and it is frequently linked with the term internal friction. E″ is sensitive to distinct types of relaxation processes, morphology, transitions, molecular motions, and other heterogeneities of the material structure. DMA aids material engineers and researchers in estimating the amount of polymer chains immobilized by the filler surface [57]. Figure 6 shows the storage modulus E’(Pa) for pure PP and for treated and untreated U(SPF/KF) /T(SPF/KF). From Table 4, the highest E’ at 20, 40, and 60 °C was recorded at 1360, 1002, and 741 MPa, respectively, which belonged to hybrid composite sample T-SP7K3. In comparison with the hybrid sample with untreated loading fiber U-SP3K7, the lowest E’ was shown at 20, 40, and 60 °C with 1200, 879, and 622 MPa, respectively. The storage modulus trend proposed a reduction in storage modulus proportional to the increase of temperature for all ratios sampled. There were no significant differences in storage modulus (E’) for any of the compositions of hybrid composite (SPF/KF/PP). It is evident that incorporation of kenaf and sugar palm fibers results in an increase in the storage modulus of the biocomposite which reveals the effective stress transfer from the fiber to the matrix at the interface.

**Figure 7:**
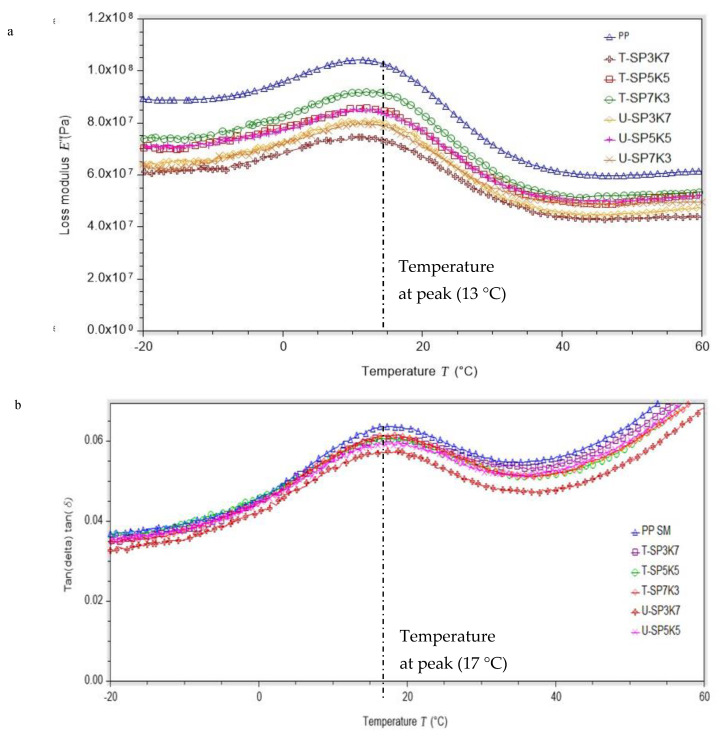
(**a**) Loss modulus at peak E” (Pa) and proportional to the temperature of varied composition of SP/K/SP hybrid composites; (**b**) Loss factor (damping) tan δ at peak in relationship with the temperature of varied compositions of SP/K/SP hybrid composite.

The loss modulus E” (Pa) was examined, which confirmed that alkalization and benzoylation on kenaf fiber aids in increasing the surface area of the fiber via the fibrillation effect, as the process splits the single-fiber bundle into small ones. At higher temperature, due to loss in stiffness of both the fiber and the matrix, the loss modulus drops. It is worth noticing that composites reinforced with benzoyl chloride and NaOH-treated fibers had a lower reduction in the value of E” when temperature was increased compared both with composites reinforced with untreated fibers and with neat residual, as shown in Figure 7a.

In particular, the loss modulus was tabulated as shown in Table 5. The loss modulus at peak (E”) was determined at peak 13 °C. The loss modulus (E”) of polypropylene (PP) was notably higher than those of the untreated and treated composites SPF/KF/PP at 105.3 MPa. Untreated hybrid composites U-SP7K3, U-SP5K5, and U-SP3K7 showed varied E” at 81.5, 80.7, and 79.8 MPa, respectively. On the other hand, treated hybrid composites T-SP7K3, T-SP5K5, and T-SP3K7 showed 71.3, 86.2, and 91.2 MPa, respectively. The higher loss modulus for the two composition ratios of treated hybrid composites T-SP5K5 and T-SP3K7 indicates that benzoylation treatment affected their mechanical properties, especially the loss modulus E” of SPF/KF/PP. Hence, this treatment escalated the effective area for the mechanical interlocking between the two phases of composites, which are fibers (kenaf and sugar palm) and polymer, and subsequently led to increased interfacial loading, which contributed to improved dynamic mechanical properties. Figure 7a shows that T-SP7K3 presented the highest loss modulus at peak, with 91.2 MPa.

In addition, Figure 7b depicts the tan δ curves of the neat PP with all composition ratios of SPF/KF/PP composites. It was observed that incorporation of kenaf and sugar palm fibers led to a pronounced decrease in the maximum value of tan δ. Neat PP showed a damping value at 0.060 Pa. For the incorporation of untreated fibers, U-SP7K3, U-SP5K5, and U-SP3K7 showed damping values of 0.056, 0.058, and 0.057 Pa, respectively. In comparison with the treated hybrid composite SPF/KF/PP, T-SP7K3, T-SP5K5, and T-SP3K7 achieved damping factors at 0.051, 0.053, and 0.052, respectively.

As observed in Figure 7b, the fibers contribution to the damping were low as compared with those of the neat PP matrix. This suggests that the combined attenuation of sugar palm and kenaf fiber reinforced composites would be primarily caused by the molecular motion of PP and the strong interaction between the fibers surface and the matrix interface. Moreover, the removal of the lignin in mercerized fibers led to a change in the extent of hydrogen-bonding, affecting the tan δ in the hybrid composites. Additionally, the width of the tan δ peak was increased in all the biocomposites; this phenomenon can be attributed to molecular relaxations taking place in the composite, which were not present in the matrix. It shows that the presence of the treated sugar palm and kenaf fibers dramatically reduced tan δ, thus indicating the presence of good adhesion, resulting in low damping. Conversely, the damping of U-SPFKF composites was found to be higher than that of neat PP resin due to weak adhesion between the hydrophilic untreated fibers and the hydrophobic polymer used as the matrix. These results also confirm the good effect of the mercerization performed on the fiber/matrix compatibility, resulting in improved stress transfer and good interfacial adhesion.

### 3.5. Thermomechanical Analysis (TMA)

Thermomechanical analysis of the pure PP as well as their hybrid composites with treated and untreated sugar palm/kenaf composites were examined at different fiber ratios. They were carried out to explore the dimensional changes or the coefficient of thermal expansion (CTE) in both regions. Due to the stretching and quenching of the composite during fabrication, internal stress in composites was created. In the testing, when an external load was applied to sample in the axial direction with temperature, the porosity of sample started to collapse or ‘shrink’, and the sample showed three phases of deformities.

The deformities began with the positive strain due to elastic creep. Near or at the T_g_ of the polymer, in between 45 and 105 °C, the creep strain was recovered, followed by shrinking. As demonstrated in Table 6, thermal expansion coefficient (CTE) after 45 °C shows pure PP with 10.23. In addition, untreated reinforced composite U-SP7K3, U-SP5K5, and U-SP3K7 were shown CTE at 1.13, 3.21, and 2.14 respectively. In addition, the treated reinforced-composite, T-SP7K3, T-SP5K5, and T-SP3K7 were shown CTE at 1.23–7.32 and 6.14. Overall, the small amount of CTE in both treated and untreated composite portrayed low CTE value as the transition state and moisture evaporation which hindered an extreme expansion. On the other hand after 105° C demonstrate U-SP7K3, U-SP5K5, and U-SP3K7 with 11.31, 24.93 and 24.74 value of CTE respectively. However, T-SP7K3, T-SP5K5, and T-SP3K7 shown a huge expansion at 12.74, 30.11, and 18.23 respectively. Due to the melting of the polymer composite, (SPF/KF/PP), CTE were shown higher at 105° 

**Figure 8:**
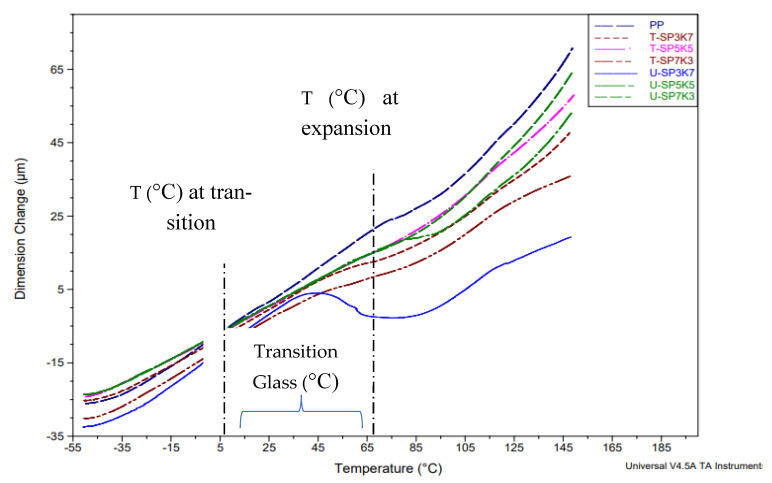
Thermogram of the dimension change in varied compositions of SP/K/SP hybrid composites.

Highly cross-linked polymers and the large number of stretched tie chains contributed to the high modulus of elasticity and reversible (recoverable) deformation [59,60]. The negative strain showed pore shrinkage, termed T_deformation_ at T_deformation_, and strain induced necking began and propagated along the drawing direction until the sample ruptured. From the TMA graphs, it may be noted that the pure PP and untreated and treated duo fiber composites showed different patterns of TMA curves. Figure 8 demonstrates that the glass transition temperature could not be detected for the neat PP, and the curves showed a steep drop for the untreated hybrid composites, which is associated with the low cross-linking in these materials compared with the treated SPF/KF/PP [61]. SPF/KF/PP composite turned out to have highly mobile materials in the rubbery stage. Benzoyl-treated sugar palm- and kenaf-reinforced composites revealed better interfacial bonding between the PP matrix, causing better surface adhesion and cross-linking in composites. Compared with the untreated fibers, the hybrid composite demonstrated a lower dimensional µm change due to the poor compatibility between untreated fibers and the polymer matrix. The curve of the T-SP7K3 hybrid composite showed rigidity in the rubbery region, which is an indication of the high degree of cross-linking of fiber [62]. The obtained results also demonstrate better mechanical properties of the T-SP7K3 hybrid composite.

## 4. Conclusions

This research investigated the thermal stability and the dynamic and thermomechanical properties of treated and untreated sugar palm- and kenaf fibers-reinforced polypropylene composites and found that composition T-SPF/KF/PP showed better thermal stability in comparison with all untreated SPF/KF/PP hybrid composite ratios. After incorporation with treated kenaf and sugar palm fiber, thermal properties of the hybrid composites were improved. The lowest weight loss wt% at T_max_ was for hybrid composite T-SP5K5, with 85.02% total residue char at 800 °C (wt%) recorded at 5.22%. The benzoylation treatment towards the fibers gave a good interfacial bonding, where the polymer acted as a barrier to prevent the degradation of the natural fibers. These results were in good agreement with the above discussion, where the effect of the benzoyl group on the surface of KF and SPF after benzoylation treatment increased the thermal stability of the composites, compared with the untreated composites. A flammability test it showed a reduction in the burning rate (mm/min) with the incorporation of treated fibers in the SPF/KF/PP hybrid composite. T-SP5K5 was determined to achieve the lowest burning rate at 22.53 (mm/min). In addition, as proved by the DSC curve, the existence of a T_g_ peak showed that the hybrid composite SPF/KF/PP in all compositions was amorphous, except for neat polypropylene, which showed no transition glass peak. The DSC curve also confirmed that the highest transition glass and melting point was for T-SP5K5, with a T_g_ at 127.63 and a T_m_ at 165.63.

Storage modulus analysis showed that hybrid composite T-SP5K5 at 20, 40, and 60 °C with 1360, 991, and 711 MPa (SPF/KF/PP) with benzoylation treatment, respectively, showed the highest storage modulus (E’). Loss modulus (E”) and damping tan δ at peak rating showed that the incorporation of fibers into a polymer restricts the mobility of the polymer chains, leading to lower flexibility, which ultimately decreases the damping characteristics. T-SP5K5 showed an E” and tan δ of 86.2 MPa and 0.0531 Pa, respectively. Furthermore, T-SP5K5 showed coefficients of thermal expansion (CTE) values after 45 and 105 °C of 7.323 and 30.11, respectively.

## Figures and Tables

**Table 1:** Compositions of sugar palm fiber, kenaf fiber, and polypropylene hybrid composite (SPF/KF/PP).

Hybrid Composites	SPF (g)	KF (g)	PP (g)	Total Weight (g)
U-SP3K7	0.6	1.4	18	20
U-SP5K5	1	1	18	20
U-SP7K3	1.4	0.6	18	20
T-SP3K7	0.6	1.4	18	20
T-SP5K5	1	1	18	20
T-SP7K3	1.4	0.6	18	20

**Table 2:** Onset temperature, maximum temperature, weight loss, and maximum temperature and residual at 800 °C, analyzed in TGA analysis.

Samples	T_on_ (°C)	T_max_ (°C)	Weight Loss at T_max_ (wt%)	Char at 800 °C (wt%)
PP	-	439	99.46	0.53
U-SP3K7	274	421.8	93.37	2.02
U-SP5K5	276.1	442.9	91.22	2.357
U-SP7K3	298.6	437.3	90.84	1.86
T-SP3K7	294.2	425.8	92.65	2.1
T-SP5K5	285.8	443.13	85.02	5.22
T-SP7K3	279.4	442.7	83.05	3.196

**Table 3:** Transition glass temperature, melting point temperature, enthalpy, and degree crystallinity of SPF/KF/PP hybrid composite.

Sample	Transition Glass Temperature (T_g_)	Melting Point Temperature (T_m_)	Enthalpy Δ*H* (J/g)	Degree Crystallinity (%)
PP	-	146.43	126.11	60.00
U-SP7K3	115.23	149.43	97.460	46.63
U-SP5K5	118.23	155.63	120.95	57.87
U-SP3K7	119.13	148.33	99.160	47.44
T-SP7K3	122.53	160.53	111.23	53.22
T-SP5K5	127.63	165.63	116.78	55.88
T-SP3K7	125.43	161.43	105.89	50.66

**Table 4:** Hybrid composite sample (SP/K/PP) with storage modulus (MPa).

	Sample with Storage Modulus (MPa)						
Temperature	PP	U-SP3K7	U-SP5K5	U-SP7K3	T-SP3K7	T-SP5K5	T-SP7K3
20 °C	1600	1290	1300	1200	1100	1360	1312
40 °C	1200	954	958	879	813	991	968
60 °C	989	701	695	622	601	711	709

**Table 5:** Loss modulus at peak (E”) (MPa) and damping at peak (Tan δ).

Sample	Loss Modulus at Peak (E″) (MPa)	Damping at Peak (Tan δ) (Pa)
PP	105.3 ± 2.16	0.0617 ± 0.012
U-SP7K3	81.5 ± 1.34	0.0564 ± 0.034
U-SP5K5	80.7 ± 1.14	0.0585 ± 0.041
U-SP3K7	79.8 ± 1.27	0.0572 ± 0.032
T-SP7K3	71.3 ± 1.62	0.0513 ± 0.023
T-SP5K5	86.2 ± 1.06	0.0531 ± 0.041
T-SP3K7	85.2 ± 1.11	0.0529 ± 0.022

**Table 6:** Thermal expansion after 45 °C and 105 °C.

Samples	Thermal Expansion (CTE) after 45 °C	Thermal Expansion (CTE) after 105 °C
PP	10.21 ± 0.12	31.23 ± 1.82
U-SP7K3	1.13 ± 0.12	11.31 ± 0.49
U-SP5K5	3.21 ± 0.14	24.93 ± 0.74
U-SP3K7	2.14 ± 0.41	24.74 ± 0.45
T-SP7K3	1.23 ± 0.53	12.74 ± 0.61
T-SP5K5	7.32± 0.81	30.11 ± 0.43
T-SP3K7	6.14 ± 0.73	18.23 ± 0.72

## Data Availability

The data presented in this study are available on request from the corresponding author.
